# Comparative Mechanical and Thermal Performance of Graphene- and Silver Nanoparticle-Reinforced PLA Fabricated by FDM 3D Printing

**DOI:** 10.3390/polym18121494

**Published:** 2026-06-14

**Authors:** Filiz Karabudak

**Affiliations:** Department of Mechanical Engineering, Faculty of Engineering and Natural Sciences, Gumushane University, 29000 Gümüşhane, Turkey; filizkarabudak@gumushane.edu.tr

**Keywords:** polylactic acid (PLA), graphene, silver nanoparticles, fused deposition modeling (FDM), polymer nanocomposites, mechanical properties

## Abstract

The increasing demand for high-performance and multifunctional polymer materials has driven interest in improving the mechanical properties of polymer components produced through additive manufacturing. This study aims to systematically investigate and comparatively evaluate the effects of low-content nanofiller incorporation on the structural, thermal, and mechanical performance of PLA-based materials produced via fused deposition modeling (FDM), with a focus on identifying filler-dependent behavior under different loading conditions. In this study, polylactic acid (PLA) composites reinforced with 0.5 wt.% graphene (Gr) and 0.5 wt.% silver (Ag) nanoparticles, added separately, were produced using fused deposition modeling (FDM) and comparatively investigated. Each nanofiller was incorporated individually into PLA-based filaments, and standard test specimens were fabricated via 3D printing. Structural, thermal, and mechanical properties were evaluated using tensile, compressive, and three-point bending tests, along with SEM, EDS, XRD, FTIR, DSC, and TGA analyses. The results showed that pure PLA exhibited typical brittle behavior and a single-stage thermal degradation profile. The tensile strength of pure PLA was 41.93 MPa, and the flexural strength was 70.76 MPa. The addition of 0.5 wt.% graphene led to noticeable improvements, particularly in flexural properties, while only a minimal (almost negligible) increase was observed in tensile strength, with tensile strength increasing to 42.24 MPa (+0.74%) and flexural strength increasing to 110.78 MPa (+56.6%). In contrast, 0.5 wt.% Ag exhibited mixed and load-dependent mechanical behavior, with slight improvements in flexural strength but reductions in tensile and compressive properties, where tensile strength decreased to 22.13 MPa (−47.2%) while flexural strength increased to 112.06 MPa (+58.3%). Structural and thermal analyses indicated that both nanofillers did not significantly alter the PLA matrix chemically, while contributing to controlled changes in material properties primarily through physical interactions. The novelty of this work lies in the comparative evaluation of graphene and silver nanoparticle reinforcement at a fixed low loading level within FDM-processed PLA, combined with a comprehensive and correlated analysis of mechanical, structural, and thermal behavior on the same specimen sets, enabling a clearer understanding of filler-dependent performance mechanisms in additively manufactured nanocomposites. Overall, it was concluded that low-rate nanofiller additions, when properly dispersed, may lead to selective improvements in the performance of PLA-based composites depending on filler type and loading mode, and show potential for advanced engineering applications such as lightweight structural components, functional sensors, and additive-manufactured parts requiring tailored mechanical performance and multifunctionality.

## 1. Introduction

Polylactic acid (PLA), a biodegradable and renewable polymer derived from lactic acid, is gaining increasing importance in terms of environmental sustainability and biomedical applications [1]. PLA’s biocompatibility and high stiffness provide advantages for implants, tissue engineering, and packaging applications, while its brittle behavior, limited elongation capacity, and relatively low thermal stability restrict its use in engineering applications with high mechanical demands [2,3]. To overcome these limitations and improve the mechanical and thermal performance of PLA, the addition of nanofillers and metal nanoparticles to polymer matrices is being extensively investigated. Graphene (Gr), a two-dimensional nanomaterial composed of single-layer carbon atoms, stands out as an effective reinforcement phase in polymer matrices due to its high elastic modulus, large surface area, and superior thermal conductivity [4,5,6]. Literature reports that graphene added in low proportions can improve the mechanical properties of polymer composites, depending on dispersion quality, interfacial adhesion, and processing conditions, and has been reported to increase tensile strength and elastic modulus and enhance thermal stability [7,8,9]. Similarly, silver nanoparticles are reported to improve functional properties in polymer matrices and, under certain conditions, may contribute to mechanical performance, although this effect is highly dependent on dispersion and aggregation state [10,11]. Furthermore, Ag nanoparticles offer significant advantages in biomedical applications by providing strong antimicrobial properties. Nanoparticle additives significantly affect not only the mechanical properties but also the crystallization behavior and thermal stability of the polymer. Graphene and certain metal nanoparticles have been reported to influence the crystallization behavior of polymer matrices through heterogeneous nucleation mechanisms, depending on particle size, surface characteristics, compatibility, and concentration, which may result in a reduction in the cold crystallization temperature (Tcc) [12]. This suggests that nanoparticles may influence crystallization behavior by altering nucleation and crystallization kinetics, as reflected in the shift in Tcc. In particular, graphene nanoplatelets may contribute to the formation of barrier-like structures that restrict heat and mass transfer, thereby enhancing thermal stability, whereas this effect is considerably less pronounced for spherical metal nanoparticles such as Ag. However, changes in the degree of crystallinity should be quantitatively confirmed by DSC or XRD analysis [13,14]. However, homogeneous distribution of additives within the matrix and strong interfacial interactions are critical; poor dispersion or agglomeration can lead to stress concentrations, causing premature fracture and performance loss [15].

In the FDM process, the crystallization behavior of polylactic acid (PLA) is influenced by multiple processing parameters, including thermal history, nozzle temperature, bed temperature, printing speed, and interlayer heat accumulation. These factors can limit the ordered arrangement of polymer chains and favor the formation of a more amorphous structure. The crystallization behavior and kinetics of PLA can be further investigated using differential scanning calorimetry (DSC), while cold crystallization processes are effectively characterized by this technique [16,17].

This holistic approach reveals in detail the strengthening effects and contributions to thermal stability provided by nanofillers in the PLA matrix even at low loadings, highlighting the potential of unique and promising materials for advanced engineering and functional applications.

The mechanical and thermal behavior of PLA-based composites is strongly governed by the type, concentration, and dispersion state of nanofillers. The incorporation of 0.5 wt.% graphene resulted in selective mechanical improvements, particularly in flexural properties, which can be attributed to its high aspect ratio; however, the limited effect on tensile strength suggests that an effective percolating network was not fully established at this low loading, meaning that the intrinsic high thermal conductivity of graphene does not directly translate into composite-scale performance improvement [18,19,20]. Moreover, the term “graphene” in this study refers to graphene nanoplatelets, whose behavior differs significantly from graphene oxide or reduced graphene oxide, and this distinction is critical for correct interpretation of results [4,21,22].

In contrast, 0.5 wt.% silver nanoparticles exhibited mixed mechanical performance, which may be associated not only with dispersion-induced stress concentrations but also with possible catalytic effects on PLA degradation under thermal or hydrolytic conditions, as reported in the literature for metal nanoparticle–polymer systems [23,24,25]. These competing mechanisms can explain the observed reductions in tensile and compressive properties despite slight improvements in flexural behavior.

Thermal and structural characterization using XRD and FTIR provides information on crystalline phases, molecular interactions, and chemical bonding, rather than direct “structural integrity,” and therefore interpretations are limited to changes in crystallinity and intermolecular structure [16,26]. Overall, the results confirm that nanofiller effects in PLA are highly dependent on dispersion quality, filler type, and loading level, and do not lead to universal improvements, but rather to selective and mode-dependent property changes in FDM-processed composites [27,28,29].

Although graphene- and silver-reinforced PLA composites have been widely investigated, most studies have focused on either graphene- or silver-containing systems separately, and direct comparisons of their individual effects at the same filler loading under identical FDM processing conditions remain limited [13,20,23,25,30,31,32,33,34]. Furthermore, the comparative influence of these nanofillers on both mechanical and thermal properties has not been sufficiently clarified, particularly in additively manufactured PLA composites where filler dispersion and processing parameters strongly affect performance [27,28,29,35,36,37]. Therefore, the present study was designed to provide a systematic comparison of graphene and silver nanoparticle reinforcements in PLA.

In this study, the nanofillers were incorporated into the PLA matrix as two separate systems to ensure clear interpretation of their individual effects. Specifically, polylactic acid (PLA) composites were prepared independently with 0.5 wt.% graphene (Gr) and 0.5 wt.% silver (Ag) nanoparticles, rather than as a hybrid or combined 0.5 wt.% total filler system. Each nanofiller was added separately into PLA-based filaments and processed under identical fused deposition modeling (FDM) conditions to enable a direct comparison of their individual influences on the structural, mechanical, and thermal properties of the resulting composites. This approach allows the distinct roles of graphene and silver nanoparticles to be evaluated without overlap or ambiguity in filler content or synergistic interactions.

## 2. Materials and Methods

This study investigates the mechanical, structural, and thermal performance of PLA-based composites fabricated via 3D printing with the incorporation of nanographene and silver nanoparticles at low loading levels (0.5 wt.% each). The selection of 0.5 wt.% nanofillers was based on previous studies reporting that low nanoparticle loadings provide an optimal balance between mechanical improvement and homogeneous dispersion in PLA-based composites [4,6,8,18,19,29,36,37]. At higher filler contents, nanoparticle agglomeration and non-uniform dispersion are more likely to occur, which can negatively affect interlayer adhesion and printing quality in FDM-processed materials [15,35,38]. Therefore, 0.5 wt.% was chosen as a representative low-content reinforcement level to minimize agglomeration effects and to systematically evaluate the influence of nanoparticle type (graphene and silver) on the mechanical, thermal, and structural behavior of FDM-produced PLA composites under comparable processing conditions [10,11,23,39]. The composite preparation, filament production, and FDM-based 3D printing processes were carefully controlled to ensure reproducibility and homogeneous specimen fabrication across all sample groups. PLA granules and nanoparticles were processed under controlled melt-mixing and extrusion conditions to obtain composite filaments, which were subsequently used to produce standard test specimens for tensile, compressive, and three-point bending analyses.

### 2.1. Materials and Production Process

Luminy LX175 polylactic acid (PLA) granules were used as the polymer matrix due to their bio-based origin and high purity. Polylactic acid (PLA) resin (Luminy^®^ LX175, TotalEnergies Corbion, Gorinchem, The Netherlands) was supplied by Filameon (Kayseri, Türkiye). According to the manufacturer’s datasheet, the PLA resin has a density of 1.24 g/cm^3^, a melt flow index (MFI) of 6 g/10 min (210 °C/2.16 kg) and 3 g/10 min (190 °C/2.16 kg), a melting temperature of 155 °C, and a glass transition temperature (Tg) of 60 °C. The material exhibits a tensile modulus of 3500 MPa, tensile strength of 45 MPa, elongation at break below 5%, and Charpy notched impact strength below 5 kJ/m^2^. The heat deflection temperature (HDT, amorphous state) is approximately 60 °C. The resin is characterized as a high-viscosity, low-flow, amorphous transparent PLA grade suitable for extrusion and additive manufacturing applications. As reinforcement phases, two nanofillers were used separately: graphene nanoplatelets (GNPs) and silver nanoparticles (AgNPs). Each nanofiller was incorporated individually into the PLA matrix at a concentration of 0.5 wt.% to form two distinct composite systems (PLA/0.5 wt.% GNP and PLA/0.5 wt.% Ag), rather than a hybrid or combined filler system.

The graphene nanofillers consisted of graphene nanoplatelets (Nanografi Nano Technology, Ankara, Türkiye), with a thickness of approximately 2–3 nm, a lateral size of approximately 1.5 µm, a specific surface area of 800 m^2^/g, and a purity of 99.9%, corresponding to a few-layer graphene structure.

The silver nanofiller consisted of 20–30 nm silver nanoparticles supplied by Sigma-Aldrich (Sigma-Aldrich, Merck, Burlington, MA, USA) Sigma-Aldrich Silver Nanoparticles, containing polyvinylpyrrolidone (PVP) as a surface stabilizer/dispersant and having a purity of 99.5% (trace metals basis), with predominantly quasi-spherical morphology.

### 2.2. Filament Extrusion

Prior to processing, PLA granules and nanofillers were dried to minimize moisture content and prevent hydrolytic degradation of PLA during melt processing. The dried PLA pellets and nanofillers were then compounded using a twin-screw extruder to ensure homogeneous dispersion of nanoparticles within the polymer matrix. The resulting composite was subsequently pelletized and reprocessed into filaments using a single-screw extruder with a 24 L/D ratio and a 30 mm screw diameter.

**Figure 1:**
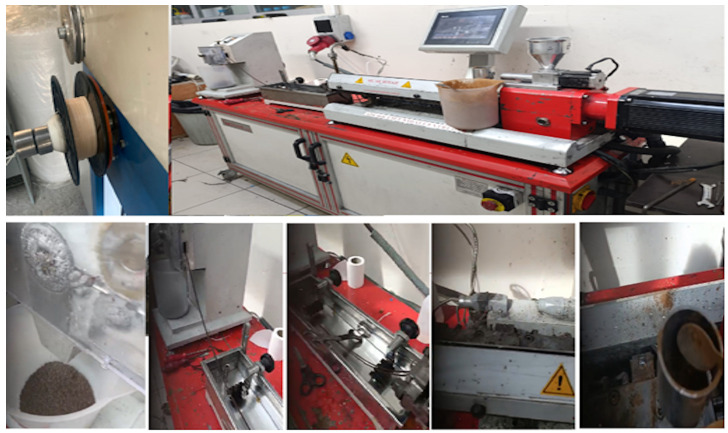
Preparation process of PLA/graphene and PLA/silver nanocomposite filaments.

The filament extrusion temperature profile was carefully optimized based on PLA rheological stability and thermal degradation behavior. The applied temperature profile was as follows: feed zone 175 °C, melting zone 191 °C, transition zone 200 °C, and die zone 207 °C. The gradual increase in temperature toward the die was selected to ensure stable melt flow, avoid premature solidification, and maintain consistent viscosity during extrusion. The screw speed was set to 23 rpm. Filaments with a controlled diameter of 1.75 mm were produced using an automatic diameter monitoring system (Figure 1).

### 2.3. Three-Dimensional Printing and Sample Preparation

The produced composite filaments were fabricated into test specimens using a Creality CR-200B Fused Deposition Modeling (FDM) 3D printer (Creality, Shenzhen, China) and Slicing software (Creality Slicer 4.8.2, Creality, Shenzhen, China). All specimens were printed using identical processing parameters to ensure comparability between groups. The main printing parameters were as follows: nozzle temperature 200 °C, bed temperature 60 °C, layer height 0.2 mm, infill density 100%, printing speed 50 mm/min, nozzle diameter 0.4 mm, and a line-type (rectilinear) infill pattern with a 45° raster orientation.

Standard test specimens were fabricated for tensile, compressive, and three-point bending tests. Tensile testing was performed according to ASTM D638-14 Type I standard [40], using specimens with a nominal thickness of 3.2 mm, which is consistent with standard dimensions commonly adopted for FDM-printed PLA composites. Tests were conducted at a crosshead speed of 5 mm/min.

**Figure 2:**
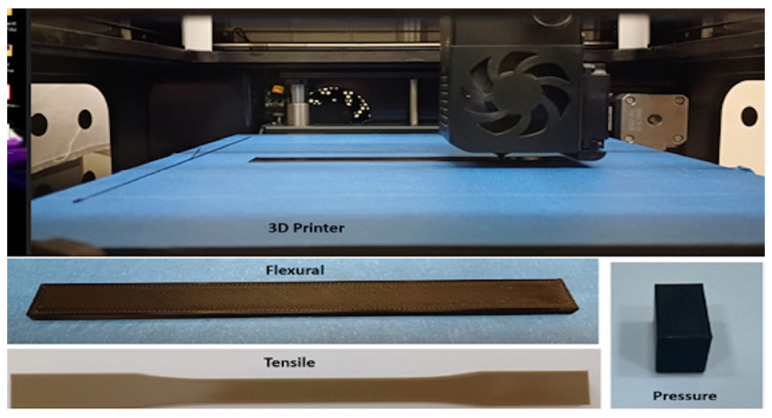
Production process of tensile, compression, and three-point bending test specimens using a 3D printer (FDM method).

Compression tests were carried out according to ASTM D695-15 standard [41] using rectangular specimens with dimensions of 12.7 × 12.7 × 25.4 mm (L × W × H), at a crosshead speed of 1.3 mm/min. Flexural properties were evaluated using three-point bending tests in accordance with ASTM D790 standard [42], on specimens with dimensions of 127 × 12.7 × 3.2 mm (L × W × T), at a crosshead speed of 2 mm/min. All tests were conducted at room temperature, and at least five specimens were tested for each condition to ensure statistical reliability using a BESMAK BMT-E Series Universal Testing Machine (Besmak, Ankara, Türkiye) (Figure 2).

## 3. Results

### 3.1. Morphological and Fracture Surface Analysis (SEM)

**Figure 3:**
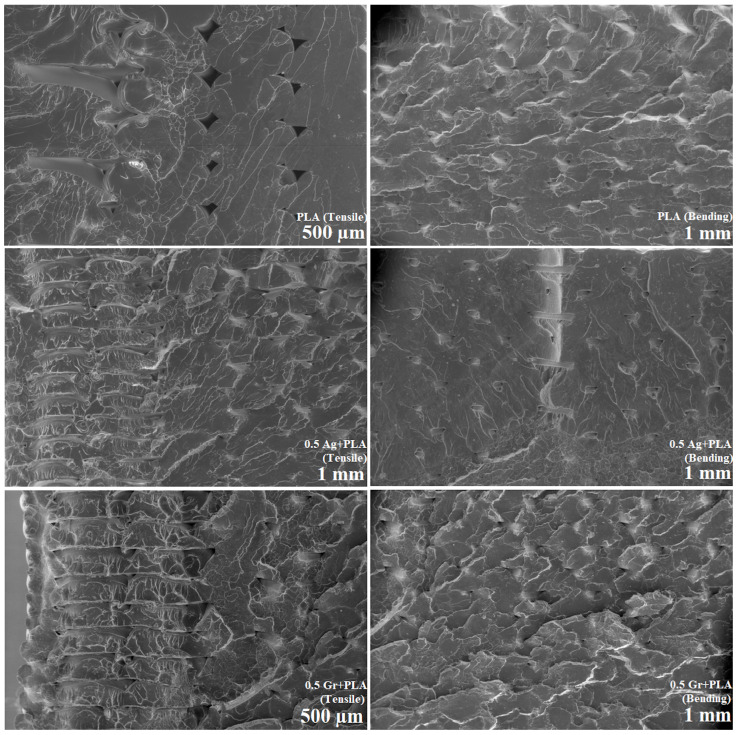
SEM images of specimens subjected to tensile and three-point bending tests.

Morphological evaluations based on SEM images presented in Figure 3 show that reinforcement elements significantly affect the load transfer mechanism and fracture behavior within the polymer matrix. The literature indicates that graphene and similar nanofillers provide effective load transfer due to their high elastic moduli, limiting crack propagation and improving mechanical properties [18,27]. While the relatively smooth fracture surfaces observed in pure PLA samples point to the brittle fracture character of the material, the increased surface irregularity and heterogeneous structure in nanofiller-reinforced samples indicate the involvement of mechanisms that hinder crack propagation. Similarly, the literature reports that nanoparticle addition improves mechanical performance and alters fracture behavior [30,43]. The crack deflection and more complex fracture surfaces observed in the bending test sections suggest that the reinforcement phases interact with crack propagation, increasing the energy absorption capacity and contributing to the material’s strength (Figure 3).

### 3.2. Energy Dispersive X-Ray Spectroscopy (EDS) Analysis

**Figure 4:**
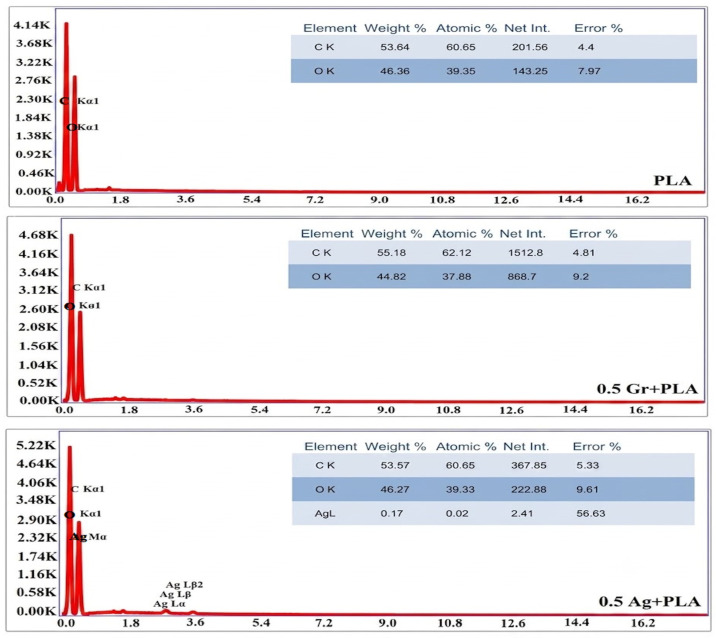
EDS analysis of neat PLA and PLA composite samples containing 0.5 wt.% graphene (Gr) and silver (Ag).

In Figure 4, the detection of only carbon (C) and oxygen (O) elements in the EDAX analysis of pure PLA is consistent with the basic chemical structure of polylactic acid [44]. This supports the chemical purity of the sample.

The similarity of carbon and oxygen ratios observed in the graphene-reinforced PLA sample can be explained by the fact that graphene is a carbon-based nanofiller added at a low concentration. Although EDAX does not detect graphene as a distinct elemental phase, variations in the local carbon signal may still occur depending on the measurement area. In addition, since EDAX is a surface-sensitive and localized technique, the measured signal can vary depending on the analyzed region and the dispersion state of the nanofiller.

In the silver (Ag)-reinforced PLA sample, the detection of low-intensity signals corresponding to silver can be attributed not only to the low concentration of nanoparticles but also to their possible agglomeration state and heterogeneous distribution within the polymer matrix. Furthermore, factors such as electron beam penetration depth and charging effects in polymeric materials may also influence the detected signal intensity in EDAX analysis.

The literature reports that graphene reinforcement improves the load transfer mechanism and enhances mechanical properties in polymer composites [19]. In this context, graphene addition contributes to the structural and mechanical properties of the composite rather than introducing a distinct elemental phase in EDAX analysis.

### 3.3. X-Ray Diffraction (XRD) Analysis

**Figure 5:**
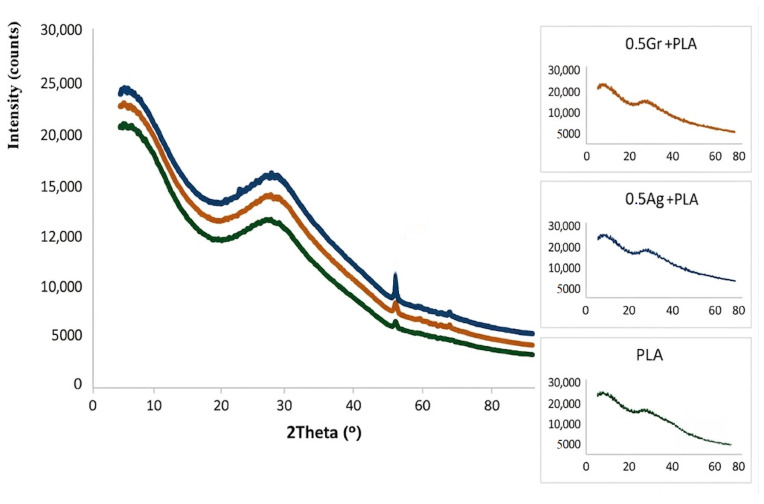
XRD analysis of neat PLA and PLA composite samples containing 0.5 wt.% graphene (Gr) and silver (Ag).

Figure 5 shows the X-ray diffraction (XRD) analysis of the crystal structure of Polylactic Acid (PLA), PLA + 0.5% Silver (Ag), and PLA + 0.5% Graphene (Gr) composite samples.

The literature reports that pure PLA exhibits weak and broad diffraction peaks in the range of approximately 2θ ≈ 16–19°, indicating a semi-crystalline structure [45]. This suggests that the PLA chains are largely in the amorphous phase and contain limited crystalline regions.

The relatively wide peaks indicate the presence of partial disorder in the polymer chains and a significant proportion of amorphous phase, a finding also reported in the literature [45,46].

In the XRD pattern of composite samples obtained by adding 0.5% by weight of Ag nanoparticles to the PLA matrix, new diffraction peaks were observed around 2θ ≈ 38°, 44°, and 64°, in addition to the characteristic peaks of PLA. These peaks correspond to the silver phase with a face-centered cubic (FCC) crystal structure, which has also been reported in the literature [23,31]. This confirms the presence of Ag nanoparticles within the composite structure and demonstrates that phase formation has occurred successfully.

When the X-ray diffraction (XRD) patterns of PLA + 0.5% graphene composites were examined, it was observed that the characteristic diffraction peaks of PLA were largely preserved. However, a weak peak was observed around 2θ ≈ 26°, corresponding to the graphitic (002) plane. This situation can be attributed to the low graphene loading and the presence of partially stacked layered structures. In the literature, it has also been reported that these peaks generally appear with weak intensity in low-rate graphene additions and their intensity increases depending on the amount of graphene [4,32,47].

### 3.4. Fourier Transform Infrared (FTIR) Analysis

**Figure 6:**
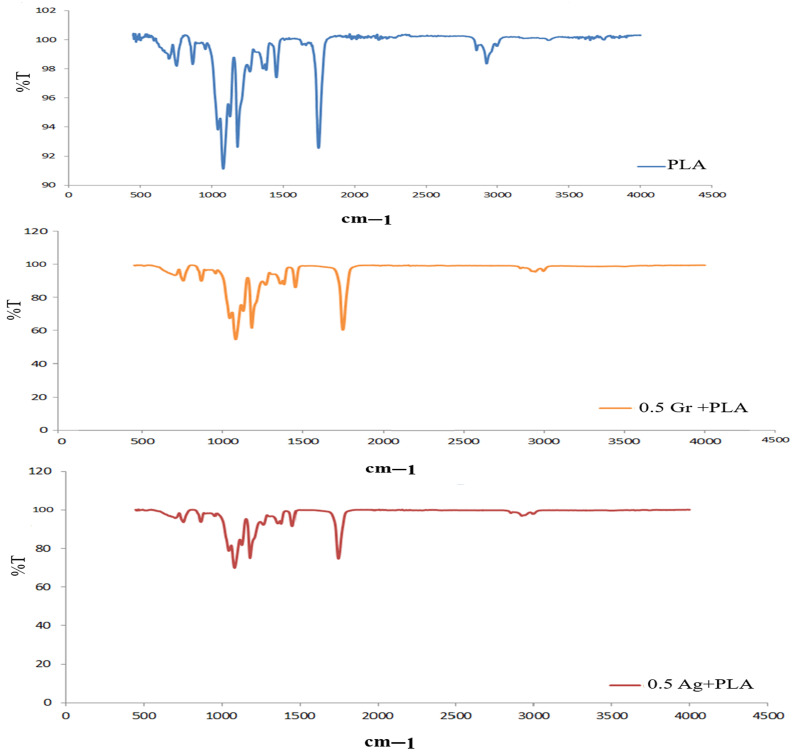
FTIR analysis of neat PLA and PLA composite samples containing 0.5 wt.% graphene (Gr) and silver (Ag).

Figure 6 shows that the FTIR spectrum of the pure PLA sample clearly exhibits characteristic peaks specific to the poly(lactic acid) structure. In particular, the strong absorption band located around 1750 cm^−1^ corresponds to ester carbonyl (C=O) stretching vibrations and is a fundamental structural indicator of PLA. In addition, C–O–C stretching vibrations were observed in the 1180–1080 cm^−1^ range and CH_3_ bending vibrations in the 1450–1380 cm^−1^ range. The obtained FTIR spectrum is consistent with PLA characteristic bands reported in the literature, indicating that the chemical structure of the material is preserved [26,44,48,49].

When the FTIR spectrum of the PLA sample with 0.5% graphene is examined, it is observed that there is no significant shift in the positions of the characteristic peaks of pure PLA, but some peak intensities change. This is attributed to the chemically inert nature of graphene and its interaction with the polymer matrix mainly through physical interactions (van der Waals forces, π–π interactions). Furthermore, due to the low graphene content, no new functional group formation was observed. Similarly, the literature reports that in graphene-reinforced polymer composites, changes in the intensities of existing peaks rather than the formation of new peaks are observed in the FT-IR spectrum [4,9,21,22,50,51].

The FTIR spectrum of the PLA sample doped with 0.5% silver (Ag) nanoparticles generally shows similarities to pure PLA; however, small intensity changes and limited band broadening were observed in some peaks. Since silver nanoparticles do not exhibit an FTIR active structure, they do not directly form characteristic peaks. However, due to weak surface interactions with the polymer matrix, small changes can occur, especially in the carbonyl (C=O) groups. This has also been reported in the literature; it is reported that Ag nanoparticles cause limited changes in the intensity and position of existing bands in polymer systems without forming new FT-IR peaks [39,52,53].

### 3.5. Thermogravimetric Analysis (TGA)

**Figure 7:**
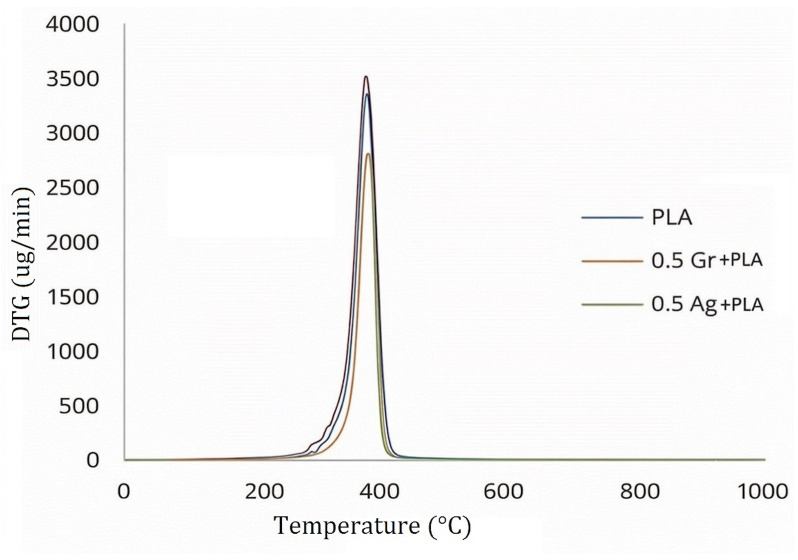
TGA of neat PLA and PLA composite samples containing 0.5 wt.% graphene (Gr) and silver (Ag).

As shown in Figure 7, DTG curves of pure PLA, PLA with 0.5% nano Gr doping, and PLA with 0.5% Ag doping were examined. When the DTG curve of the pure PLA sample was evaluated, a single, sharp degradation peak was observed in the range of approximately 350–380 °C. This indicates that PLA typically has a single-step thermal degradation mechanism. In the literature, it has been reported that the thermal degradation of PLA occurs via a random chain scission mechanism of ester bonds and therefore exhibits a single DTG peak [3,24,54]. The obtained results are consistent with the characteristic thermal degradation behavior of pure PLA.

When the DTG curve of the PLA sample with 0.5% graphene reinforcement is examined, it is observed that the maximum degradation temperature (Tmax) shifts slightly to higher temperatures compared to pure PLA, and a change in peak intensity is observed. This behavior may be attributed to the presence of graphene, which can act as a physical barrier within the polymer matrix and may partially restrict the diffusion of volatile degradation products. In addition, graphene may contribute to improved thermal resistance through enhanced heat dissipation within the composite; however, it should be noted that thermal conductivity does not directly translate into thermal stability improvements. Therefore, the observed shift is considered to arise mainly from barrier and interfacial effects rather than thermal conductivity alone.

In contrast, the PLA sample containing 0.5% Ag nanoparticles also shows a slight change in the degradation profile and a marginal shift in Tmax. This behavior may be related to dispersion effects and interfacial interactions within the matrix. In addition, it should be noted that metallic nanoparticles such as Ag may also influence the thermal degradation behavior of PLA, potentially including catalytic effects under certain conditions, although such effects are highly dependent on particle dispersion, surface chemistry, and concentration. Therefore, the overall thermal response results from competing stabilizing and destabilizing mechanisms within the composite system, as also discussed in the literature on polymer thermal degradation mechanisms [24,54].

### 3.6. Differential Scanning Calorimetry (DSC) Analysis

**Figure 8:**
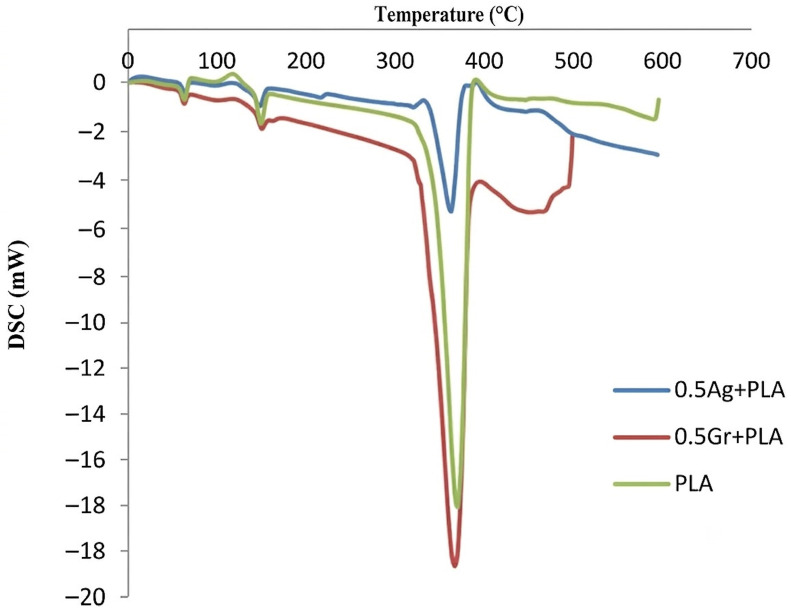
DSC analysis of neat PLA and PLA composite samples containing 0.5 wt.% graphene (Gr) and silver (Ag).

The DSC curves given in Figure 8 show the thermal behavior of pure PLA, 0.5% Ag + PLA, and 0.5% Gr + PLA samples produced by the Fused Deposition Modeling (FDM) method. In the FDM process, extruding the material in the melt and rapidly cooling it limits the formation of a regular crystalline structure of the chains and results in a more amorphous structure. This is confirmed by the appearance of distinct cold crystallization (Tcc) peaks in the DSC curves [38]. It has been reported in the literature that at high cooling rates, PLA does not have sufficient time for crystallization and forms a largely amorphous structure, while delayed crystallization during heating appears as an exothermic peak (Tcc) [16,17].

In addition, the nanoparticles added to the composite structure have a significant effect on the crystallization behavior. It has been reported that these additives can influence crystallization kinetics by promoting heterogeneous nucleation and leading to changes in the cold crystallization temperature (Tcc) [12]. In this context, the Tcc changes observed in Ag and graphene-doped PLA samples can be attributed to the possible nucleation effect of nanoparticles.

The glass transition temperature (Tg), observed in all samples at approximately 55–65 °C, indicates that the effect of filler additives on polymer chain mobility remains limited. The literature also reports that low-rate graphene addition generally does not cause a significant change in the Tg value of PLA [13].

When the cold crystallization region is examined, it is observed that the Tcc peak shifts to lower temperatures and becomes more pronounced in the graphene-reinforced (0.5 Gr + PLA) sample. This can be attributed to graphene acting as a heterogeneous nucleating agent, accelerating the crystallization kinetics. The literature also reports increased crystallization rates and lower crystal formation in graphene-reinforced PLA composites [33].

When evaluated in terms of melting behavior (Tm), the higher melting enthalpy (ΔHm) observed in the graphene-doped sample indicates an increased degree of crystallinity. This can be explained by graphene promoting more regular packing of polymer chains due to its high surface area [13]. In contrast, the wider and relatively lower intensity melting peak in the Ag-doped sample may indicate a more heterogeneous distribution in the crystal structure.

In mechanical tests, at least three different samples were tested for each sample group, and the average values of the obtained data were taken into consideration. In this way, the effect of the additive phase on the PLA matrix was revealed in a more reliable and representative manner.

### 3.7. Tensile Test Analysis

**Figure 9:**
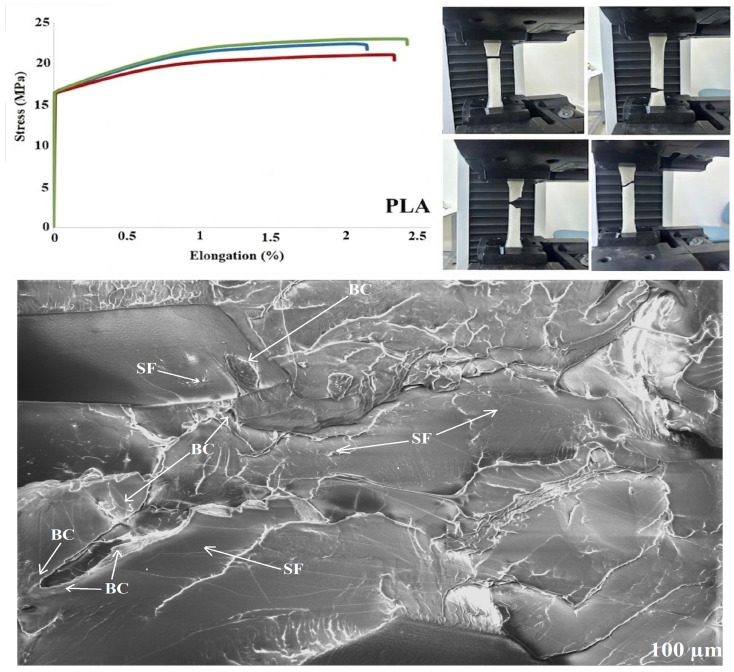
Tensile test curves and corresponding SEM images of neat PLA. * SF: Smooth Fracture, BC: Brittle Cracking.

**Figure 10:**
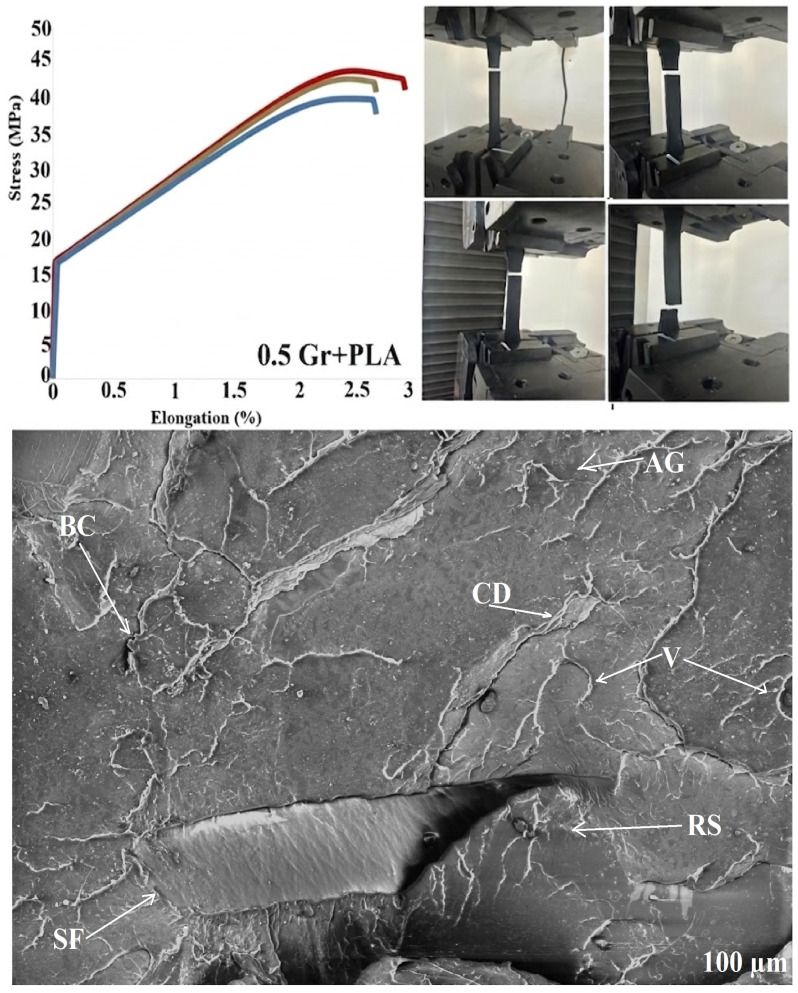
Tensile test curves and corresponding SEM images of neat PLA samples containing 0.5 wt.% graphene (Gr). * SF: Smooth Fracture, BC: Brittle Cracking, AG: Agglomeration, RS: Rough Surface, CD: Crack Deviation.

**Figure 11:**
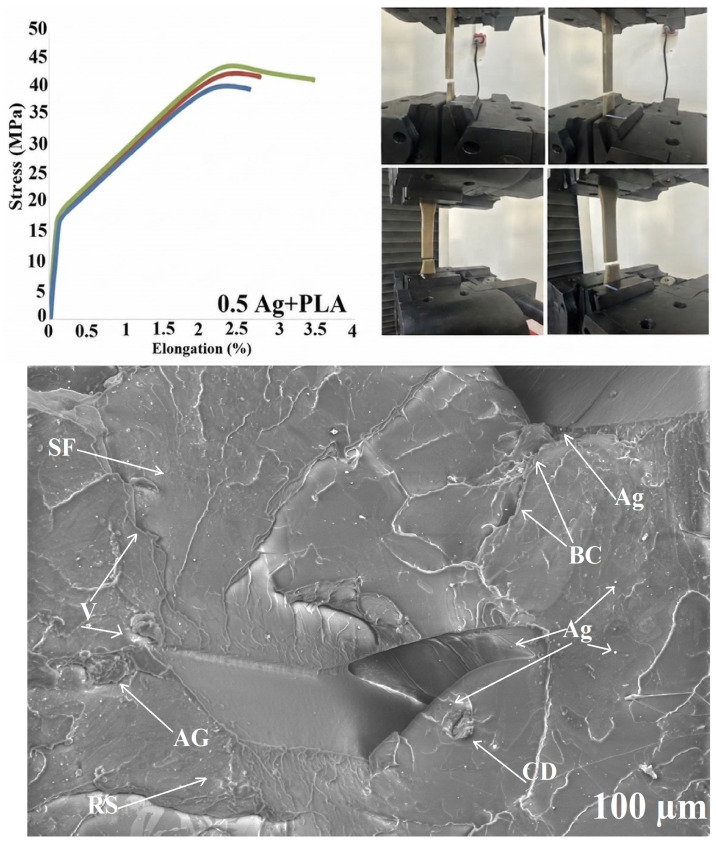
Tensile test curves and corresponding SEM images of neat PLA samples containing 0.5 wt.% silver (Ag). * SF: Smooth Fracture, BC: Brittle Cracking, AG: Agglomeration, RS: Rough Surface, CD: Crack Deviation, V: Voids, Ag: Silver.

Figure 9, Figure 10 and Figure 11 present a comparative analysis of the tensile test results of PLA composite samples reinforced with 0.5 wt.% nanographene (Gr) and PLA composite samples reinforced with 0.5 wt.% silver (Ag), prepared separately. When mechanical tests and SEM analyses are evaluated together, it is seen that nanofiller reinforcement has a significant effect on the mechanical performance of the PLA matrix. This improvement is generally attributed to the fact that filler phases improve load transfer within the polymer matrix, delay crack propagation, and create a more rigid structure during deformation. These mechanisms are consistent with the nanocomposite behaviors reported in the literature [18,43,55]. Furthermore, the more tortuous and rough fracture surfaces observed in SEM images for graphene-reinforced samples are consistent with the slight improvement in tensile strength, suggesting that crack deflection and crack-bridging mechanisms contributed to increased resistance against crack propagation [5,7,18,28]. In contrast, the presence of heterogeneous fracture regions, voids, and particle clusters in Ag-containing samples correlates with the significant reduction in tensile strength, indicating ineffective stress transfer and localized stress concentration caused by nanoparticle agglomeration [15,25,29,34,36,37]. The agglomeration tendency of Ag nanoparticles can be attributed to their high surface energy and strong particle–particle interactions, which promote clustering within the PLA matrix during melt processing [36,37,39].

SEM analyses support this mechanical behavior at the microstructural level. The flatter and more homogeneous fracture surfaces observed in pure PLA samples (SF—Smooth Fracture) indicate brittle fracture characteristics (BC) and limited energy absorption capacity. In contrast, graphene and silver-reinforced samples showed rougher and more heterogeneous fracture surfaces (RS), with a change in crack propagation direction and increased energy absorption at the fracture surface. The crack deviations (CD) observed particularly in the graphene-reinforced structure indicate increased energy consumption during fracture [18,28]. The relatively more homogeneous morphology observed in graphene-reinforced samples may be associated with the use of low graphene loading (0.5 wt.%), controlled melt-mixing conditions, and twin-screw extrusion processing, which helped improve graphene dispersion within the PLA matrix and reduced excessive agglomeration formation [4,6,8,18,29].

When evaluating the mechanical test results, the tensile strength and elongation of the pure PLA sample were determined to be 41.93 MPa and 2.94%, respectively. These values, which are below the typical PLA tensile strength range reported in the literature, can be attributed to the production conditions, the weakness of interlayer bonding due to the FDM process, and microstructural heterogeneity [1,35]. Since all specimens were fabricated using identical FDM printing parameters and raster orientation, the differences in mechanical performance are primarily attributed to the effects of nanofiller addition and the resulting microstructural changes rather than variations in processing conditions [35].

For the samples reinforced with 0.5 wt.% graphene, the tensile strength and elongation at break were measured as 42.24 MPa and 2.70%, respectively. Compared to pure PLA, this corresponds to an increase of approximately 0.74% in tensile strength and a decrease of 8.16% in elongation at break. This situation can be explained by the fact that graphene, thanks to its high surface area (RS) and mechanical strength, partially increases the load-carrying capacity within the polymer matrix and limits chain mobility (CD). However, it is considered that the effective strengthening effect remains limited due to the low dopant ratio and possible dispersion limitations [4,18,56].

In contrast, the samples containing 0.5 wt.% Ag exhibited a tensile strength of 22.13 MPa and an elongation at break of 2.30%. Compared to pure PLA, this represents a decrease of approximately 47.2% in tensile strength and 21.8% in elongation at break. This significant decrease can be explained by the fact that silver nanoparticles are not homogeneously distributed within the matrix, exhibit a tendency towards agglomeration (AG), and that effective load transfer cannot be achieved due to weak interfacial interactions and voids (V—Voids). In addition, such heterogeneous distributions lead to local stress concentrations and microcrack formation under tension, triggering premature fracture [25,34].

Overall, the results show that graphene addition can improve mechanical performance in the PLA matrix to a limited extent, while Ag addition can lead to a decrease in mechanical properties due to dispersion and interface problems.

### 3.8. Compression Test Analysis

**Figure 12:**
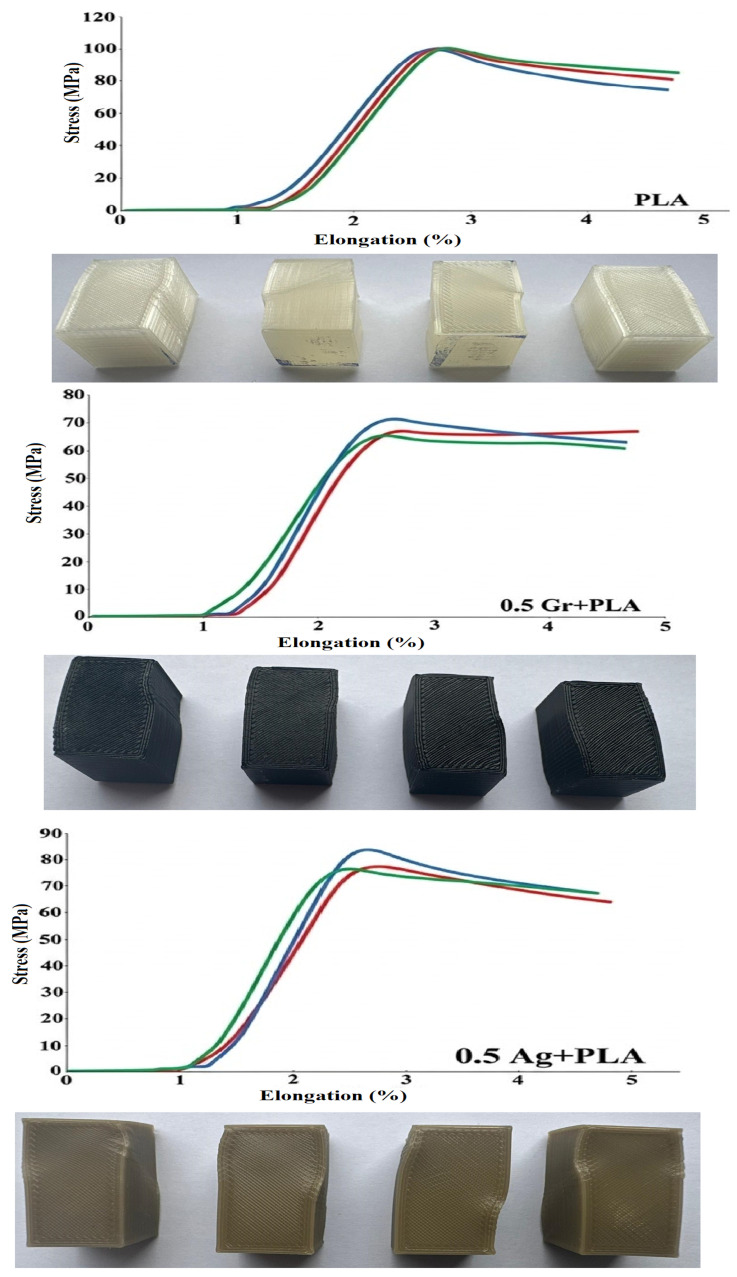
Compression test curves and specimen images of neat PLA and PLA samples containing 0.5 wt.% graphene (Gr) and silver (Ag).

In Figure 12, the findings clearly demonstrate the effect of nanofiller reinforcement on 3D-printed PLA matrix at both macro- and micro-scales. The addition of 0.5 wt.% graphene (Gr) and 0.5 wt.% silver (Ag), incorporated separately into PLA matrices, shows significant effects on mechanical strength, energy absorption capacity, and deformation behavior. This improvement is based on the fact that nanofillers strengthen weak interlayer bonds by bridging between polymer chains, increasing load transfer efficiency. This mechanism has been widely reported in the literature [4,18]. Furthermore, the SEM observations are consistent with the compression test results, indicating that the microstructural features of the reinforced samples directly influenced their deformation and fracture behavior under compressive loading. In particular, the relatively more homogeneous morphology observed in graphene-containing samples may have contributed to improved stress transfer and delayed crack propagation, whereas heterogeneous regions and particle agglomerations in Ag-containing samples likely promoted localized stress concentration and premature microcrack initiation [5,15,18,29,36,37].

When compression test results and SEM analyses are evaluated together, it is seen that the reinforced samples exhibit a more homogeneous stress distribution under load and brittle fracture is delayed. In samples with a cubic geometry, it was observed that deformation occurred more controllably and crack propagation was partially limited compared to pure PLA. This behavior can be attributed to the presence of nanofiller supporting structural integrity at the micro-scale. The improved graphene distribution can be associated with the use of low filler loading (0.5 wt.%), controlled melt-processing conditions, and extrusion-based mixing, which may have reduced excessive nanoparticle agglomeration and enhanced interfacial interactions within the PLA matrix [4,6,8,18,29].

SEM images confirm these behavioral differences observed in mechanical tests at the microstructural level. While smoother and more homogeneous fracture surfaces were observed in pure PLA samples, brittle fracture behavior, where cracks propagate along layer interfaces, was dominant. In contrast, rougher and more irregular fracture surfaces were formed in graphene and silver-doped samples, and it was observed that graphene nanoplates, in particular, activated the crack deflection mechanism by deflecting the crack propagation direction [7,18]. In Ag-reinforced samples, however, the presence of particle clusters and heterogeneous fracture regions suggests that silver nanoparticles tended to agglomerate within the PLA matrix. This behavior can be attributed to the high surface energy of Ag nanoparticles and strong nanoparticle–nanoparticle interactions, which promote clustering during melt processing and reduce the efficiency of stress transfer under compressive loading [34,36,37,39].

According to the compression test results, the maximum compressive strength for the pure PLA sample was determined as 100.11 MPa. This value is consistent with the high rigidity and limited plastic deformation characteristics of PLA reported in the literature [1,2]. The semi-crystalline structure of PLA contributes to brittle deformation behavior by limiting chain mobility [57]. In samples with 0.5% silver (Ag) additive, the compressive strength decreased significantly to 67.83 MPa. This corresponds to a decrease of approximately −32.3% compared to pure PLA. This decrease can be explained by the non-homogeneous distribution of silver nanoparticles within the matrix, the formation of agglomerations, and the inability to achieve effective load transfer due to weak interfacial interactions. Such heterogeneities lead to local stress concentrations and early microcracking under tension, reducing mechanical strength [29,36,37].

In graphene-added samples, the compressive strength was determined to be 79.24 MPa, exhibiting a higher performance compared to Ag-added samples. This corresponds to a decrease of approximately −20.8% compared to pure PLA. This is related to graphene’s high elastic modulus and large surface area, which allows for more effective load transfer within the polymer matrix. Furthermore, the presence of graphene enhances structural integrity by strengthening interfacial interactions [4,5,6].

In general, the results show that graphene addition has a mechanical performance-enhancing effect in the PLA matrix, while silver nanoparticle addition can lead to a decrease in mechanical properties due to dispersion and interfacial problems.

### 3.9. Three-Point Bending Test Analysis

**Figure 13:**
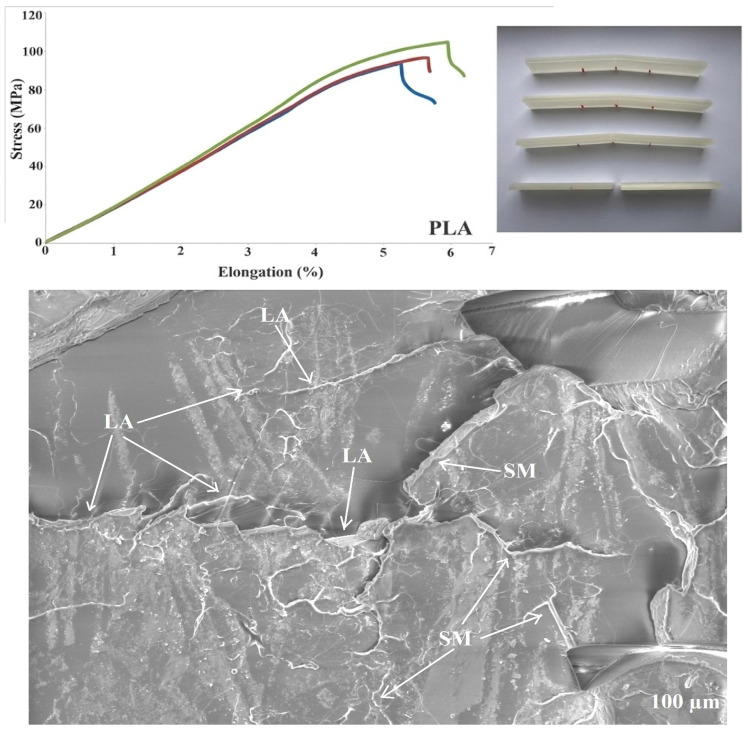
Three-point bending test curves and SEM images of neat PLA. * LA: Layer Adhesion Failure, SM: Smooth Matrix.

**Figure 14:**
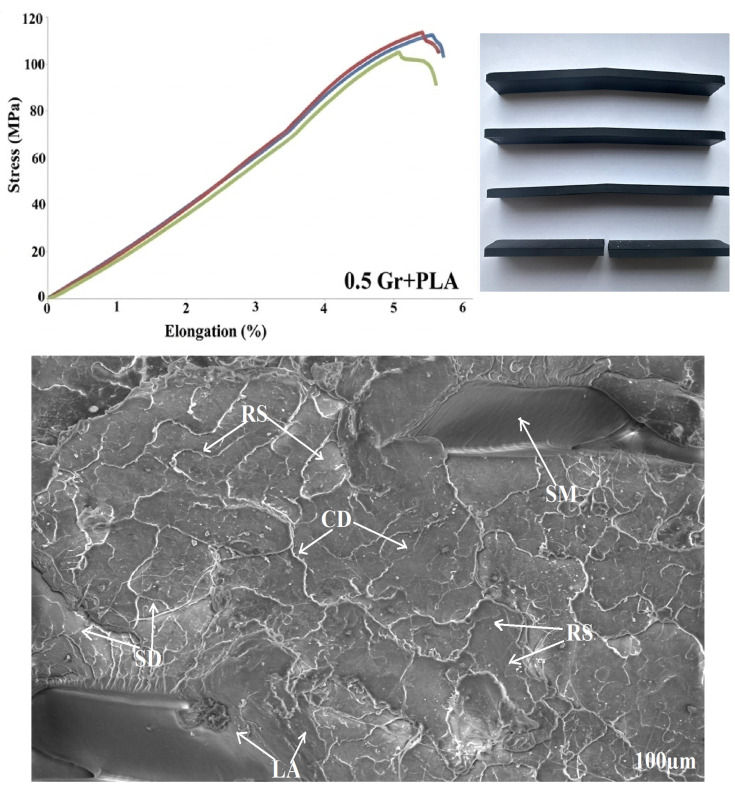
Three-point bending test curves and SEM images of PLA samples containing 0.5 wt.% graphene (Gr). * LA: Layer Adhesion Failure, SM: Smooth Matrix, RS: Rough Surface, CD: Crack Deflection, SD: Ductile Deformation.

**Figure 15:**
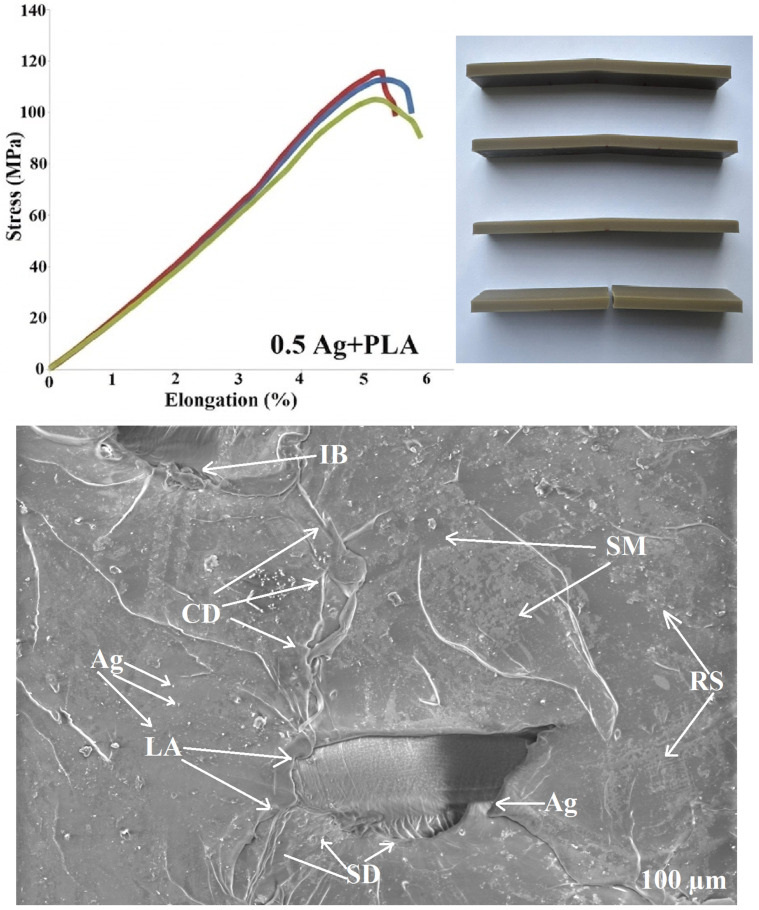
Three-point bending test curves and SEM images of PLA samples containing 0.5 wt.% silver (Ag). * LA: Layer Adhesion Failure, SM: Smooth Matrix, RS: Rough Surface, CD: Crack Deflection, IB: Interfacial Bonding, SD: Ductile Deformation, Ag: Silver.

In Figure 13, Figure 14 and Figure 15, three-point bending test results and accompanying SEM analyses show that nanoparticle reinforcement affects interlayer interactions (IB) and fracture morphology in 3D-printed PLA-based parts. In general, nanofiller addition contributed to the partial improvement of weak regions between layers (LA) and may facilitate more efficient stress transfer within the polymer matrix. This behavior is mainly associated with improved interfacial interactions and localized reinforcement effects rather than a fully uniform load transfer mechanism. Similar trends have also been reported in the literature [4,18,27]. In addition, SEM observations suggested that graphene nanoplatelets were distributed more uniformly within the PLA matrix compared to Ag nanoparticles, which may be related to the high surface area and planar morphology of graphene promoting better interaction with polymer chains during melt mixing and extrusion [4,5,6,18,21]. The improved graphene distribution may also contribute to stronger interlayer bonding and more effective crack deflection mechanisms in FDM-produced structures [18,35,38].

SEM investigations provide qualitative support for these observations. In pure PLA samples, relatively smooth fracture surfaces were observed, indicating brittle fracture behavior dominated by crack propagation along layer interfaces (LA). In contrast, graphene- and silver-doped samples exhibited rougher and more irregular fracture surfaces (RS), suggesting increased energy dissipation during fracture. In particular, graphene nanoplatelets may contribute to crack deflection (CD) and tortuous crack paths, which can increase fracture resistance [7,18]. On the other hand, Ag-containing samples exhibited localized particle clusters and heterogeneous regions in SEM images, indicating a tendency toward nanoparticle agglomeration within the PLA matrix. This behavior can be associated with the high surface energy of metallic nanoparticles and insufficient interfacial compatibility with the polymer matrix, which may promote particle–particle interactions instead of homogeneous dispersion [29,34,36,37,39]. Such agglomerated regions may act as local stress concentration points and weaken interlayer adhesion under flexural loading conditions [15,36,38].

The flexural test results indicated that the pure PLA sample had a flexural strength of 70.76 MPa and an elongation of 6.06%. These results indicate that PLA exhibits relatively high rigidity but limited deformation capacity due to its intrinsic semi-crystalline structure [1,2].

In samples with 0.5% graphene (Gr) addition, the flexural strength increased to 110.78 MPa. This corresponds to an increase of approximately +56.6% compared to pure PLA. This improvement can be attributed to the high aspect ratio of graphene, its large surface area, and its ability to enhance interfacial interactions and stress transfer within the PLA matrix. However, the extent of reinforcement is strongly dependent on dispersion quality and the formation of an effective reinforcing network. Agglomeration or non-uniform distribution may significantly reduce the expected reinforcement efficiency [4,19,29,36,37]. The relatively rough and tortuous fracture morphology observed in SEM images of graphene-reinforced samples supports this interpretation, suggesting that improved graphene distribution contributed to crack pinning, crack deflection, and delayed fracture propagation during bending deformation [7,18,28].

In samples with 0.5% silver (Ag) addition, the flexural strength was measured as 112.06 MPa, which is close to that of graphene-reinforced samples. This corresponds to an increase of approximately +58.3% compared to pure PLA. This similarity can be explained by a combination of local stress redistribution, defect-induced toughening effects, and interfacial interactions between Ag nanoparticles and the PLA matrix. Unlike graphene, Ag nanoparticles do not inherently induce ductility in PLA; therefore, the observed mechanical response is more likely related to microstructural heterogeneity and dispersion state rather than true plasticization. It is well documented that the mechanical performance of metal nanoparticle–polymer composites is highly dependent on dispersion uniformity, agglomeration tendency, and interfacial bonding characteristics [10,11,29,36,37].

Overall, the results indicate that graphene addition provides a more consistent reinforcing effect in the PLA matrix, while silver nanoparticle addition can lead to comparable flexural performance under favorable dispersion and interaction conditions, although the underlying strengthening mechanisms differ.

## 4. Discussion

This study investigated the effects of separately reinforcing PLA matrix composites produced by the FDM method with 0.5 wt.% graphene (Gr) and 0.5 wt.% silver (Ag) nanoparticles on their mechanical, thermal, and structural properties. The results show that even low nanoparticle addition altered the microstructure and macro-scale behavior of PLA. However, the observed behaviors may also be influenced by factors such as nanoparticle dispersion quality and the anisotropic layer-by-layer structure inherent to the FDM process [35,38]. Similar effects of interlayer-dependent mechanical behavior and anisotropic fracture characteristics in FDM-processed PLA composites have also been widely reported in previous studies [35,38,40].

SEM analyses revealed that the layered and brittle fracture behavior observed in pure PLA was transformed into a rougher and more irregular fracture morphology with the addition of nanoparticles. The increased prominence of crack deflection and bridging mechanisms, particularly in graphene-containing samples, suggests enhanced resistance to crack propagation during fracture. This behavior is related to graphene’s high mechanical strength and large surface area, which may facilitate more effective load transfer between the PLA matrix and the nanofiller, and has been similarly reported in the literature [7,8,18].

The mechanical test results are consistent with the microstructural observations. While a limited increase in tensile strength was observed with graphene addition, a significant improvement in flexural strength from 70.76 MPa to 110.78 MPa (+56.55%) was obtained. This behavior may be associated with the different stress states generated under tensile and flexural loading conditions. In bending, the material is simultaneously subjected to tensile and compressive stresses, and graphene may contribute to delaying crack initiation and propagation under these combined loading conditions. Improvements in mechanical performance in PLA/graphene composites have been similarly reported in the literature [13,33]. The improved flexural response may also be associated with the ability of graphene nanoplatelets to partially strengthen interlayer regions and distribute local stresses more effectively during bending deformation [18,27,28].

In contrast, the significant decrease in tensile strength (22.13 MPa) in the Ag-added sample can be explained by the non-homogeneous distribution of nanoparticles and possible agglomeration formation. Previous studies have also emphasized that nanoparticle agglomeration leads to stress concentrations, causing mechanical weakening [36,37]. However, the slight increase in flexural strength suggests that Ag nanoparticles may influence the deformation behavior differently under bending loads, despite their adverse effect on tensile performance. The agglomeration tendency of Ag nanoparticles may be related to their high surface energy and insufficient compatibility with the PLA matrix, promoting particle clustering during melt processing and filament extrusion [29,34,36,37,39]. The mechanical test results are consistent with the microstructural observations (Table 1).

XRD and FTIR analyses showed that the chemical structure of PLA was preserved in all samples and that nanoparticle addition did not lead to the formation of distinct new chemical bonds detectable by FTIR. This indicates that the interactions are predominantly physical, although weak interfacial interactions such as dipole–dipole interactions or adsorption effects may still be present [20,21]. This observation is consistent with previous studies reporting that graphene- and metal nanoparticle-reinforced PLA systems generally exhibit physical rather than covalent interactions within the polymer matrix [20,21,27].

Furthermore, it has been reported in the literature that graphene can affect the crystal structure by acting as a nucleating agent in polymer systems [13,27].

DSC results confirmed the presence of cold crystallization behavior in all samples and showed that PLA maintained its semi-crystalline structure depending on the FDM production process. Graphene addition accelerated crystallization kinetics, causing a decrease in the Tcc value. This behavior may be attributed to the heterogeneous nucleation effect of graphene, which can promote crystal formation by providing additional nucleation sites. However, crystallization behavior is also influenced by factors such as polymer chain mobility and confinement effects within the matrix [17,45]. Ag addition showed this effect to a more limited extent. The relatively weaker influence of Ag nanoparticles on crystallization behavior may be associated with their lower aspect ratio and less effective nucleation capability compared to graphene nanoplatelets [12,27].

TGA analyses showed that graphene addition increased thermal stability and delayed the decomposition temperature. This effect can be attributed to graphene’s barrier properties, which limit the diffusion of volatile degradation products. Similarly, increased thermal stability in graphene-based polymer nanocomposites has been widely reported in the literature [8,56]. The effect of Ag addition on thermal stability, however, remained more limited. The enhanced thermal resistance observed in graphene-containing samples may also be associated with the formation of partially interconnected barrier-like structures restricting heat and mass transfer during degradation [14,56].

Overall, the results show that the nanoparticle type is a determining factor in the performance of PLA matrix composites. Graphene addition provided more consistent improvements in both mechanical and thermal properties, while Ag nanoparticles exhibited selective improvements depending on the loading mode. This difference is directly related to the dispersion characteristics, interface interactions, and agglomeration tendency of the nanoparticles [29,36]. These findings are in agreement with previous studies emphasizing that nanoparticle dispersion quality and interfacial compatibility are among the most critical parameters governing the overall performance of polymer nanocomposites produced by additive manufacturing techniques [27,28,29,38].

In addition to the mechanical and thermal enhancements observed in this study, material extrusion (FDM)-processed polymer nanocomposites have been widely reported to exhibit multifunctional capabilities, including structural health monitoring, thermal management, and sensing applications. For instance, CNT-based polymer nanocomposites have been demonstrated for real-time structural health monitoring through electrical response changes under mechanical loading [58]. Similarly, FDM-fabricated PLA/graphene-based systems have been engineered to form continuous conductive networks, significantly improving thermal conductivity and enabling advanced heat dissipation and functional material design strategies [59]. These studies highlight the broader potential of FDM-produced polymer nanocomposites beyond mechanical reinforcement, particularly in the development of multifunctional engineering materials.

## 5. Conclusions

The present study demonstrated that the incorporation of graphene and Ag nanoparticles affected the structural, mechanical, and thermal performance of FDM-produced PLA composites in different ways depending on nanoparticle type and dispersion behavior. SEM observations revealed notable changes in fracture morphology, with graphene-containing samples exhibiting more evident crack deflection and bridging features, suggesting improved fracture resistance. Mechanical testing showed that 0.5 wt.% graphene provided the most favorable combination of properties, increasing the tensile strength from 41.93 MPa to 42.24 MPa (+0.74%) and the flexural strength from 70.76 MPa to 110.78 MPa (+56.55%). In contrast, although the addition of 0.5 wt.% Ag increased the flexural strength to 112.06 MPa (+58.3%), it reduced the tensile strength to 22.13 MPa (−47.2%), indicating load-dependent behavior with contrasting effects that may be associated with nanoparticle agglomeration, local void formation, weak interfacial adhesion, and stress concentrations. Structural and thermal analyses confirmed that the fundamental chemical structure of PLA was maintained after nanoparticle incorporation, while FT-IR results did not reveal distinct new chemical bonds and therefore suggest predominantly physical interactions between the matrix and nanofillers. Furthermore, graphene promoted crystallization and improved thermal stability more effectively than Ag. Overall, the findings indicate that graphene showed a more favorable combination of mechanical and thermal performance under the investigated conditions, whereas the performance of Ag-containing composites appears to be strongly dependent on dispersion quality and interfacial characteristics. These results highlight the importance of nanoparticle distribution and processing optimization in achieving reliable multifunctional PLA-based nanocomposites.

## Figures and Tables

**Table 1:** Tensile, flexural, and compressive properties of PLA and PLA-based nanocomposites produced via FDM.

Material	Tensile Strength (MPa)	Change (%)	Flexural Strength (MPa)	Change (%)	Compressive Strength (MPa)	Change (%)
PLA (Pure)	41.93	—	70.76	—	100.11	—
PLA + 0.5 wt.% Graphene	42.24	+0.74%	110.78	+56.55%	79.24	−20.8%
PLA + 0.5 wt.% Ag	22.13	−47.2%	112.06	+58.3%	67.83	−32.3%

## Data Availability

The data supporting the findings of this study are available within the manuscript. No additional datasets were generated or analyzed.
